# Rationale and Design of the Impact of Air Pollution Due to DESERT Dust in Patients with HEART Failure (DESERT HEART)

**DOI:** 10.3390/jcm12154990

**Published:** 2023-07-29

**Authors:** Alberto Domínguez-Rodríguez, Pablo Avanzas, Néstor Báez-Ferrer, Pedro Abreu-González, Sergio Rodríguez, Sebastian Matos-Castro, Daniel Hernández-Vaquero

**Affiliations:** 1 Servicio de Cardiología, Hospital Universitario de Canarias, Ofra S/N La Cuesta, E-38410 Tenerife, Spain; nestor.baez@hotmail.com; 2 Facultad de Ciencias de la Salud, Universidad Europea de Canarias, 38300 Tenerife, Spain; sebastianmanuel.matos@universidadeuropea.es; 3 CIBER de Enfermedades Cardiovasculares (CIBERCV), 28029 Madrid, Spain; avanzaspablo@uniovi.es; 4 Área del Corazón, Hospital Universitario Central de Asturias (Oviedo), Instituto de Investigación Sanitaria del Principado de Asturias, 33011 Oviedo, Spain; dhvaquero@gmail.com; 5 Departamento de Medicina, Universidad de Oviedo, 33012 Oviedo, Spain; 6 Departamento de Fisiología, Facultad de Medicina, Universidad de La Laguna, 38200 Tenerife, Spain; pabreugonzalez@gmail.com; 7 Instituto de Productos Naturales y Agrobiología (IPNA), CSIC, 38206 Tenerife, Spain; sergio.rodriguez@csic.es

**Keywords:** air pollution, desert dust, chronic heart failure, airway inflammation, oxidative stress, design study, pulmonary inflammation, haze

## Abstract

Aims: The main objective of this study is to determine whether exposure to Saharan dust causes airway inflammation and oxidative stress in patients with stable chronic heart failure (HF) and a left ventricular ejection fraction of less than 40%. Methods: A longitudinal study design is used, involving the inclusion of 40 patients with stable chronic HF and a left ventricular ejection fraction of less than 40%. Four sputum samplings will be taken from each patient, with one sampling taken each week over four consecutive weeks. The sputum samples will be used to analyze the degree of inflammation and oxidative stress. Air quality monitoring stations will be used to analyze the particulate matter (PM) exposure of each patient. The intrusion of desert dust will be identified using meteorological models. There will be 160 scheduled samplings in 40 patients with chronic HF. Mixed regression models will be used to assess the influence of the concentrations of PM (from the episodes of desert dust) upon the airway inflammation and oxidative stress markers. Conclusion: The results of this study will test the hypothesis that exposure to high concentrations of Saharan dust affects the normal function of the respiratory epithelium due to the imbalance between the production of free radicals and antioxidant enzymes, thus causing increased pulmonary inflammation and oxidative stress in patients with HF that in turn may facilitate decompensations of their background disease condition.

## 1. Introduction

The natural history of heart failure (HF) is characterized by acute decompensation episodes, which are the leading cause of hospitalization among patients older than 65 years of age [1].The main reason why patients with acute HF seek urgent medical care is increasing signs and symptoms of congestion [1]. Although some patients are admitted with a clear correctable trigger, in many patients there is no clear precipitating factor. Triggering factors that result in increased fluid retention and the appearance of symptoms can be detected in patients with chronic HF [2]. Among the different triggering factors, air pollution has emerged in recent years as a harmful element for the health of patients with HF [3,4].

Although air pollution consists of a complex mixture of compounds in the form of gases and particulate matter (PM), there is more evidence supporting PM as the main cause of cardiovascular effects [3]. PM is classified according to the aerodynamic diameters of its components into fractions: PM_10_ (“thoracic particles”, <10 μm), PM_2.5–10_ (“coarse particles”, 2.5–10 μm), PM_2.5_ (“fine particles”, <2.5 μm), and UFP (“ultrafine particles”, <0.1 μm) [5].

Many epidemiological studies have demonstrated a clear association between exposure to air pollutants and cardiovascular health problems [3]. Many of these studies have been made in urban areas of Europe, North America, and Asia, where the population is exposed to numerous gaseous (nitrogen oxides, ozone, sulfur dioxide, among others) and particulate pollutants (hydrocarbons, soot, metals, sulfates and nitrates, etc.) largely related to combustion phenomena (diesel- and gasoline-driven vehicles, the generation of electricity, industrial processes, etc.) [6].

**Figure 1:**
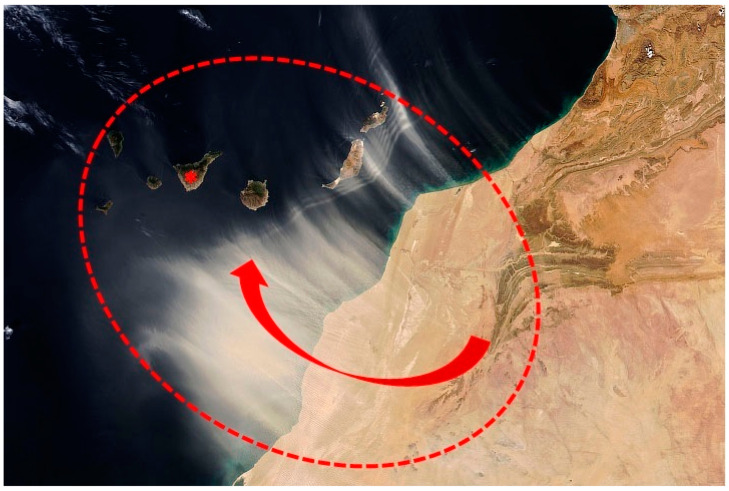
Massive dust storm over the Canary Islands due to the Saharan dust belt (inside red dashed circle). The red arrow represents the motion of the dust from theAfrican continent. (*): represents the island of Tenerife in the Canary Islands archipelago.

In recent years, the scientific community has shown interest in the possible health effects of exposure to PMx contained in desert dust [7]—a phenomenon also known as haze in meteorological terms. The potential variability in the chemical composition of the PMx must be taken into account when interpreting the results of studies on health effects based on registries of the levels of PMx. Most studies on cardiovascular mortality and PMx have been carried out in medium latitude urban areas where the population is mainly exposed to PMx from combustion sources (particularly fine and ultrafine carbon-based particles such as hydrocarbons and soot, the burning of biomass, and industrial emissions), with only sporadic episodes of exposure to desert dust, at concentrations of 10–60 µg/m^3^ [8]. In this regard, due to their proximity to the dry zones of northern Africa, the Canary Islands constitute a “research laboratory” for studying the effect of desert dust (haze) upon human health (Figure 1).

Previous epidemiological studies have evidenced increases in morbidity and mortality during desert dust episodes in southern Europe [7] and Asia [9]. Our research group conducted the first study in Spain to determine whether exposure to Saharan dust episodes is predictive of hospital admissions among patients with acute HF. The study was carried out in the Emergency Care Department of Hospital Universitario de Canarias (Canary Islands, Spain) and showed the presence of high Saharan dust concentrations (PM_10_: 50–200 µg/m^3^) to be correlated with hospital admission among patients with acute HF (odds ratio [OR] = 2.36; 95% confidence interval [95% CI] 1.21–4.58; *p* = 0.01) [10]. Recently, a meta-analysis demonstrated that exposure to PM from desert dust has been associated with increased cardiovascular mortality, both on the day of exposure and on the previous day [11].

The pathophysiological mechanism whereby exposure to desert dust can induce systemic cardiovascular effects in chronic HF patients is not clear. The lack of studies examining the degree of airway inflammation and oxidative stress in chronic HF patients exposed to desert dust episodes highlights the need for a design study addressing this highly relevant clinical question.

## 2. Methods

### 2.1. Study Design

In the healthcare setting, and more specifically in the emergency care departments of the Canary Islands, it is well known that there is an increase in hospital admissions due to respiratory problems on days characterized by high Saharan dust concentrations [12]. In the last 25 years, the analysis of sputum has become a safe, robust, and valid tool for evaluating airway inflammation in both the research and the clinical setting. The study of this biological fluid is reproducible, with scant variability [13].

**Figure 2:**
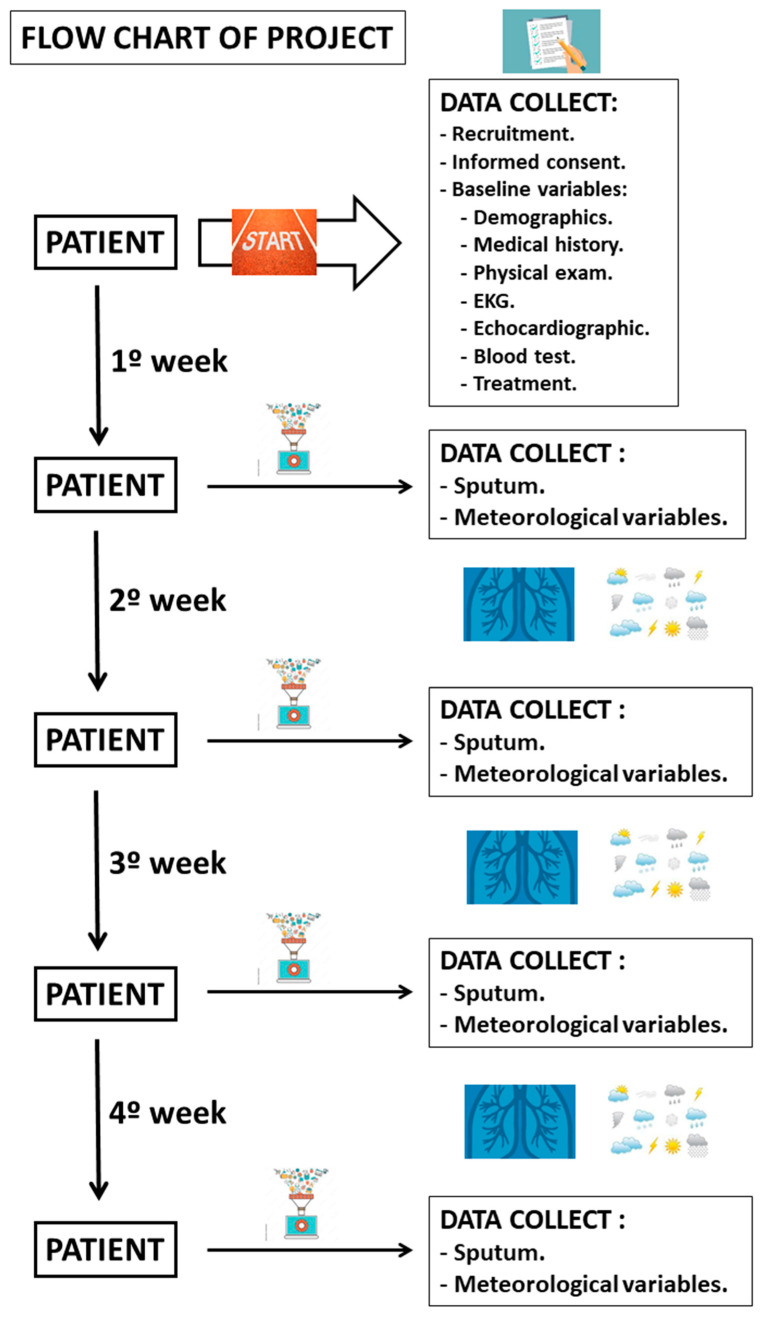
DESERT HEART study flow chart.

The impact of air pollution due to desert dust in patients with heart failure (DESERT HEART) is a study of longitudinal design involving consecutive patients observed with HF and reduced ventricular ejection fraction in specialized HF clinics. The study flow chart is shown in Figure 2. Each patient will carry a portable device with an internal temperature and humidity sensor designed to measure the concentration of particles in the air. The device is to be carried for four consecutive weeks. Moreover, four sputum samplings will be made per patient, with one sampling taken each week on the same day of the week over four consecutive weeks. The PMx measurements entered in the online database will coincide with each sputum sampling each week over four consecutive weeks.

### 2.2. Study Objectives and Hypothesis

When the dust waves reach an urban area, the population becomes exposed to a mixture of desert dust and local urban and regional pollution, i.e., PM_10_ is the sum of PM_10-dust_ + PM_10-non-dust_ (particles generated by industry, motor vehicles, combustion, etc.). In the present study, our hypothesis is that the exposure to high concentrations of Saharan dust (PM_10_ > 50 µg/m^3^) affects the normal function of the respiratory epithelium due to the imbalance between the production of free radicals and antioxidant enzymes, subsequently causing increased pulmonary inflammation and oxidative stress in patients with HF that in turn may facilitate decompensation of the background disease condition.

The primary endpoint is to determine whether or not exposure to Saharan dust implies increased airway inflammation and oxidative stress in chronic HF patients. The secondary endpoint is to determine whether desert dust exposure below the threshold of PM_10_ > 50 µg/m^3^ (the threshold recommended by the World Health Organization) results in a negative effect upon the bronchial lumen in chronic HF patients or gives rise to increased airway inflammation and oxidative stress.

### 2.3. Study Population

The present study will include outpatients of both sexes diagnosed with HF and presenting a left ventricular ejection fraction < 40% without age limitations. The selected patients will present New York Heart Association (NYHA) functional class I symptoms, with optimized treatment according to the criterion of the supervising physician and based on the current clinical guidelines. In accordance with the objectives of the study, patients with the following characteristics will not be included: (a) active smokers, (b) ex-smokers, (c) passive exposure to tobacco smoke, (d) chronic obstructive pulmonary disease, (e) asthma, (f) lung emphysema, (g) acute lung or bronchial disease, (h) occupational lung disease, (i) acute infectious or viral disease.

The longitudinal study will allow us to follow our subjects in real time. This means we can better establish the real sequence of events, allowing for insight into cause-and-effect relationships. We will select patients presenting HF at NYHA stage I. Each patient included will be evaluated every week for 4 consecutive weeks. When the patient comes to the outpatient clinic, a sputum sample will be taken, meteorological variables will be collected, and the patient will be clinically evaluated to confirm that they are in NHYA stage I. Each patient will undergo a clinical history, physical examination, chest X-ray, and blood test to determine BNP at each visit. Each patient will have a portable device that will measure dust particles during the 4 consecutive weeks.

### 2.4. Study Variables

The prospectively collected data will be entered in an online database. The following baseline variables will be recorded at the time of patient inclusion in the study:

- Demographic data: age and sex.
- Clinical history: arterial hypertension, diabetes mellitus, hyperlipidemia, and the type of dilated cardiomyopathy (idiopathic or ischemic).
- Physical examination: blood pressure, heart rate, weight, and height.
- Electrocardiographic parameters: cardiac rhythm.
- Echocardiographic parameters: left ventricular ejection fraction (%), the type of diastolic dysfunction, left ventricle size, left atrium size, the presence of grade II-III mitral valve insufficiency.
- Laboratory blood test parameters: hemoglobin (g/dL), NT-proBNP (ng/L), glomerular filtration rate (mL/min/1.73 m^2^).
- Treatments: angiotensin converting enzyme inhibitors, sacubitril–valsartan, betablockers, aldosterone antagonists, ivabradine, automated defibrillator, resynchronization therapy, sodium–glucose cotransporter inhibitors
- Variables referring to inflammation of the bronchial lumen: transforming growth factor-beta 1 (TGF-β1) and hydroxyproline.
- Variables referring to oxidative stress of the bronchial lumen: malondialdehyde (MDA).
- Variables to assess markers of systemic inflammation and endothelial dysfunction.
- Individual exposure variables: analysis of exposure for each patient corresponding to PM_10_, PM_2.5–10_, and PM_2.5_, as well as analysis of gaseous pollutants.
- Meteorological parameters: temperature, relative humidity, and wind speed.
- Environmental–individual variables: (a) season of the year; (b) place of residence (rural or urban); (c) approximate distance from the home of the patient to the monitoring station.
- Presence of Saharan dust: dichotomic variable referring to the mean daily concentration of PM_10_ > 50 μg/m^3^.

### 2.5. Processing of Sputum Samples

The selection of patients, as well as the collection of the sputum samples and the recording of “air data”, will be made during the two desert dust seasons: the winter hazes (November–March) and the summer hazes (July–August).

Four sputum samplings will be taken from each patient. One sampling will be taken each week over four consecutive weeks. If the first sample is obtained on a Monday, the remaining three samples will be collected on the next three Mondays. In this way we intend to avoid the possible effects of season, long-term tendencies, or day of the week on the airway inflammation and oxidative stress markers.

Sputum induction will be performed using a hypertonic saline spray (3% NaCl). The sputum samples will be stored at −70 °C until they are processed for analysis. Each sample will be thawed and washed twice with a cold saline solution before then being dried on filter paper and accurately weighed. Each sample will be homogenized in a cold saline solution using ultrasound (100 W for 5 s). The homogenate will be filtered through a sterile nylon dressing and centrifuged at 1000× *g* for 5 min at 4 °C. The supernatant aliquots will be stored at −70 °C until analysis.

The TGF-β1 concentrations will be assayed using a commercial ELISA kit following the instructions of the manufacturer (IBL International GmbH, Hamburg, Germany). The hydroxyproline concentrations will be assayed using the collagen determination spectrophotometry method of Reddy and Enwemeka [14].The MDA concentrations will be assayed using the spectrophotometry technique of Kikugawa et al. [15].

### 2.6. Sample Size Calculation

In longitudinal studies with mixed regression models, prior calculation of the sample size is complex, not standardized, and requires important prior knowledge [16,17]. No study has analyzed the impact of Saharan desert dust on airway inflammation and oxidative stress markers in chronic HF patients. The calculation of approximate sample size has been based on previous studies that successfully analyzed the influence of environmental parameters upon cardiovascular and endothelial inflammation. Mirowsky et al., in a study of 117 samplings in 13 patients (4–10 samples per patient), recorded the harmful effect of ozone upon a series of endothelial damage and inflammatory markers [18]. Recently, Lin et al., in a study of 78 samples in 26 individuals (3 samples per individual), showed that travelling from a city with low pollution levels to a city with intense pollution resulted in an increase in different proinflammatory and pro-oxidant markers in blood and urine [19]. Thus, the present study involving 160 scheduled samplings in 40 patients with chronic HF will probably have the statistical power needed to determine the influence of the concentrations of PM_10_ (from the episodes of desert dust) upon the airway inflammation and oxidative stress markers.

### 2.7. Statistical Analysis

Based on repeated measures, each subject will be compared with themselves, thus serving as their own control and limiting the need to adjust for individual variables. Since the usual individual variables and comorbidities (age, sex, diabetes, etc.) do not vary significantly from one day to another, these parameters have no relevant impact upon changes in the levels of airway inflammation and oxidative stress markers within the same individual. On the other hand, the collection of four sputum samples on the same day of the week over four consecutive weeks seeks to control for possible changes in the inflammation and oxidative stress markers produced by seasonality, long term tendencies, or the day of the week.

The influence of the PM_10_ concentrations during desert dust episodes upon the bronchial inflammation and oxidative stress markers will be studied using mixed regression models while controlling for the following environmental parameters: PM_2.5–10_, PM_2.5_, NO_2_, SO_2_, O_3_, temperature, and relative humidity. The maximum model will be constructed with PM_10_, the pollutants, the aforementioned environmental particles, and the second-order interactions between PM_10_ and the rest of the environmental variables. The interactions will be assessed jointly and will be eliminated from the model if no statistical significance is observed. We also will take into account the possibility that the impact of the environmental parameters may be delayed by a few days. In this respect, we will examine the effect of the environmental parameters upon the inflammation and oxidative stress markers on the same day (0 day lag), the delayed effect (1–4 day lag), and the average of the same day and the previous four. The individual variables will not be entered in the regression models since, as mentioned above, we will use repeated measurements from each patient, thus eliminating the need to control for individual variables [18,20]. All the variables will undergo log transformation before analysis, and estimation of the effect will be expressed as the percentage change in biological marker level with respect to the average due to an increase in the exposure variable from percentile 25 to percentile 75 (interquartile range [IQR]). In other words, we will estimate the percentage change in the biological marker through its 95% CI, assuming that the PM_10_ concentration during the desert dust episodes varies from a relatively low value (percentile 25) to a relatively high value (percentile 75) [18]. All the analyses will be made using STATA v.15^®^ (StataCorp., College Station, TX, USA).

## 3. Discussion

**Figure 3:**
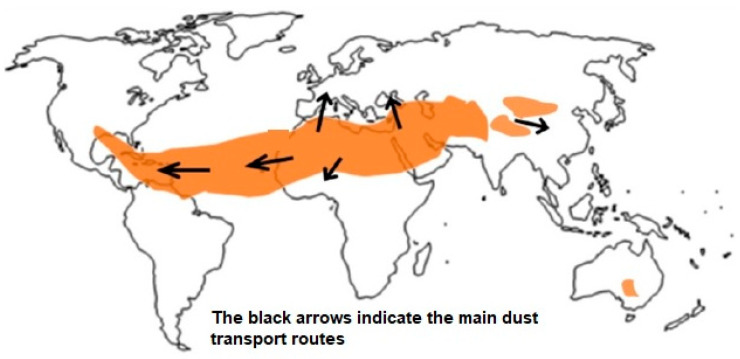
Dust belt.

The main desert dust-emitting regions are found in the so-called dust belt, which extends through northern Africa, the Middle East and inner Asia [21] (Figure 3). The greatest sources are located in northern Africa, representing 50–70% of global dust emissions. From the meteorological and climatic perspective, the Canary Islands are characterized by two desert dust seasons: the winter hazes and the summer hazes.

The winter season extends from November to March, when the anticyclone conditions in northern Africa give rise to the emission of dust at low levels of the atmosphere (generally below 500 m altitude [22]) with PM_10_ and PM_2.5_ concentrations that may be very high (>500 µg/m^3^ for PM_10_ and >180 µg/m^3^ in the case of PM_2.5_) in turn, the summer dust season (July and August) is linked to the Saharan thermal descent and the consequent African monsoons. In these cases, the dust reaches the Canary Islands over a wider atmospheric range and reaches an altitude of 5 km [23] during heat episodes (heat waves in some cases), with potential concentrations of >100 µg/m^3^ for PM_10_ and >30 µg/m^3^ in the case of PM_2.5_. The spring is characterized by sporadic episodes generally related to the penetration of showers in the Mediterranean, which induce strong winds in northern Africa and the emission of dust towards the Atlantic [24].

## 4. Conclusions

DESERT HEART is a study which will test the hypothesis that exposure to high concentrations of Saharan dust affects the normal function of the respiratory epithelium due to the imbalance between the production of free radicals and antioxidant enzymes, thus causing increased pulmonary inflammation and oxidative stress in patients with HF that in turn may facilitate decompensations of their background disease condition.

## Data Availability

The data presented in this study are available on request from the corresponding author.
